# Context-Dependent and Context-Independent Effects of D1 Receptor Antagonism in the Basolateral and Central Amygdala during Cocaine Self-Administration

**DOI:** 10.1523/ENEURO.0203-19.2019

**Published:** 2019-08-13

**Authors:** Earnest S. Kim, K. Matthew Lattal

**Affiliations:** Department of Behavioral Neuroscience, Oregon Health & Science University, Portland, OR 97239-3098

**Keywords:** amygdala, cocaine, contextual renewal, extinction, SCH 23390, self-administration

## Abstract

One way that drugs of abuse perturb the dopamine system is by triggering large amounts of extracellular dopamine to efflux into limbic regions. The basolateral (BLA) and central (CeA) nuclei of the amygdala have been shown to play distinct roles in value representation of primary and conditioned reward. However, the precise role of dopaminergic receptors in the BLA and the CeA during reward-related behaviors remains to be determined. Here we investigate the effects of dopamine D1 receptor blockade in the BLA and the CeA during asymptotic performance of cocaine self-administration and in a novel application of contextual renewal under continued access conditions. After more than three weeks of chained seek-take self-administration of cocaine, male Long Evans rats were given a bilateral intra-BLA or intra-CeA infusion of the D1 antagonist SCH-23390 (2 µg/0.3 µl) for multiple days. Intra-BLA D1 receptor blockade before, but not after the self-administration session, gradually suppressed drug seeking and taking responses and persisted with a change in context with continued D1 blockade. In contrast, intra-CeA D1 receptor blockade caused a rapid reduction in self-administration that showed renewal with a change in context with continued D1 blockade. Further, conditioned place aversion developed with intra-BLA but not intra-CeA infusions. Collectively, these results demonstrate that dopamine D1 receptors in the BLA and CeA both contribute to drug seeking and taking, but may do so through distinct mechanisms.

## Significance Statement

Drugs of abuse perturb the dopamine system by triggering large amounts of dopamine to efflux into limbic regions. The basolateral (BLA) and central (CeA) nuclei of the amygdala play distinct roles in value representation of primary and conditioned reward. However, the precise role of dopaminergic receptors in the BLA and the CeA during reward-related behaviors remains to be determined. Here we show that D1 antagonism in the BLA and CeA suppressed cocaine seeking and taking behavior, but only the BLA effect resisted contextual renewal. This study provides evidence for a novel method to weaken the effects of cocaine itself during active drug self-administration, and thereby provides insights into possible therapeutic interventions that may help prevent relapse.

## Introduction

Drug-seeking is thought to be maintained by a combination of positive and negative reinforcement processes that balance the appetitive and aversive effects of a drug. Although the amygdala is commonly associated with aversive processes, many studies have shown the importance of the amygdala in appetitive processes (Gallagher et al., 1990; Johnson et al., 2009). Appetitive and aversive outcomes, as well as cues that predict them, evoke dopamine efflux to the amygdala where D1- and D2-like receptors are expressed (Hurd et al., 1997; Guarraci et al., 1999, 2000; Weiss et al., 2000; Fotros et al., 2013; Abraham et al., 2014; Janak and Tye, 2015).

Much of what is known about the role of dopamine receptors in the amygdala in cocaine reward comes from models of relapse in which a self-administration phase is followed by instrumental extinction and a subsequent test for relapse under extinction conditions (See et al., 2003; Marchant et al., 2013). In this model, D1 receptor blockade has been shown to weaken cue but not drug-prime induced reinstatement of drug-seeking behavior (See et al., 2001). Although these studies have demonstrated dopaminergic function in the amygdala to be critical for conditioned stimuli to direct appropriate instrumental responses, most of these studies use extinction procedures where drug paired environments and responses are extinguished a number of days before an unextinguished drug paired conditioned stimulus is presented.

Recent approaches to drug-seeking and relapse incorporate conflict-based models in which aversive contingencies are added after a self-administration phase in which animals have continued drug access. In these models, inactivation of the basolateral (BLA) and the central nucleus of the amygdala (CeA) were found to increase context-induced relapse after punishment, but not after extinction. However, inactivation of the BLA, but not the CeA, increased context-induced relapse in the acquisition context after punishment in an alternate context (Pelloux et al., 2018). These results highlight that the mechanisms that weaken maintained responding for drug reward are different depending on whether the behavioral approach involves removal of the drug reward or insertion of an aversive contingency during continued drug access conditions (Marchant et al., 2019). Given these findings, how dopaminergic function in the BLA and CeA can differentially modulate active drug self-administration where an animal has continued drug access remains unresolved.

In the following experiments, we evaluate the effects of delivery of the dopamine D1 receptor antagonist SCH 23390 to the BLA or CeA under continued drug access conditions. We find that antagonism of these receptors over multiple days results in rapid (in the case of CeA) or gradual (in the case of BLA) weakening of cocaine self-administration. The suppression of responding in the BLA is reversed when the antagonist is removed and the context is changed, but it persists across contexts when the antagonist continues to be delivered. In contrast, the rapid reduction in responding with CeA infusion is reversed with a context change even when the antagonist continues to be delivered in the new context.

## Materials and Methods

### Subjects

Male Long Evan rats (Charles River Laboratories) initially weighing 300–350 g (seven to eight weeks) were housed individually under a reverse 12/12 h light/dark cycle (light off at 7:00 A.M.; all experiments occurred during the dark phase). After surgery and a recovery period, rats were food restricted and maintained at 350–400 g during the course of the experiments. All experiments were performed according approved protocols by the Oregon Health & Science University Institutional Animal Care and Use Committee. A total of 87 rats were used, with eight rats excluded for failure of catheter patency and five rats were excluded for misplaced cannulas.

### Intracranial and catheter surgery

Rats were given an intraperitoneal injection of an induction anesthetic mixture of ketamine/xylazine (94 mg/kg, 6.2 mg/kg) and maintained with isoflurane (1%). Rats were implanted with chronic guide cannulas (22 gauge; Plastics One) in the CeA (AP: –2.1 mm, ML: ± 4.0 mm, DV: –6.2 mm) or the BLA (AP: –2.5 mm, ML: ± 5.2 mm, DV: –7.4 mm). Guide cannulae were positioned ∼0.75 mm above target sites using a Paxinos and Watson (1998) and permanently attached using six stainless-steel screws (MiniTaps) and dental cement. Dummy cannulas (0.014–0.36 mm, Plastics One) were inserted into guide cannulas until microinjection days. Rats were then implanted with an intravenous catheter constructed of SILASTIC laboratory tubing (Dow Corning) and inserted into the right jugular vein anchored with silk suture tied to a silicone gel ball (Silicon; DAP). The SILASTIC tubing ran subcutaneously and exited near the mid-scapular region and attached to a stainless-steel cannula (Plastics One Inc.) screwed into a custom-made backpack (Flashforge Creator Pro3D) designed in Tinkercad and printed using Simplify 3D. Following implantation, the catheter was injected daily with sulfamethoxazole trimethoprim (TEVA) and heparin to maintain catheter patency and reduce infection. Rats were given 5–7 d to recover and were given ad libitum food and water before self-administration behavior. Catheter patency was confirmed with sodium brevital (2 mg/kg) as needed.

### Self-administration (Experiments 1–3)

#### Apparatus

Contexts A and B: two separate contexts (A and B) were used where the floor, the visual cues, and the spatial location of half of the chambers in each room were altered. Context A consisted of a grid floor of 19 large (4.8 mm) diameter bars spaced 15.6 mm apart, a clear Plexiglas back wall, and was located in a different location (vertically and horizontally) within each testing room. Context B consisted of a grid floor of 26 smaller (3.2-mm) diameter bars spaced 8.0 mm apart, a black and white striped back wall, and in a distinct location (vertically and horizontally) within each testing room. Both sets of chambers had a house light and two retractable levers with a cue light above each lever.

#### General behavioral methods

Behavioral experiments were performed in the operant chambers described above (Med Associates). One lever was paired with a cocaine infusion (take lever) while the other lever was designated as a seek lever (counterbalanced). All experiments began with levers retracted and the house light extinguished. Rats were food restricted to maintain starting body weight and dosage during the course of the experiment.

#### Self-administration training of a cocaine seek-take chain schedule of reinforcement

The seek-take procedure was modified from procedures described previously (Chen et al., 2013; Alaghband et al., 2018). Briefly, self-administration training began after 1 min with an insertion of a take lever and the illumination of the house light. One lever press on the take lever resulted in a single 5-s cocaine infusion (0.45–0.48 mg kg^−1^ at a rate of 0.088 ml/5 s), retraction of the take lever, illumination of a cue light above the take lever for 5 s, and extinction of the house light. Following a 30-s inter-trial interval, another trial was initiated with an insertion of the take lever and illumination of the house light. After stable responding of 30 infusions across two consecutive days, a seek lever was inserted with the take lever retracted and the house light illuminated. One press on the seek lever resulted in the retraction of the seek lever and insertion of the take lever. One press on the take lever resulted in a single cocaine infusion and illumination of the cue light above the take lever. After stable responding of 30 infusions across two consecutive days, a variable interval schedule on the seek lever of VI2, VI3, VI5, and VI10 seconds was introduced each consecutive day. Training sessions terminated after either 30 infusions or 4 h. Rats were maintained on the VI 10-FR1 schedule of reinforcement throughout the rest of each experiment, except where noted below.

#### Experiment 1A: Effects of BLA SCH 23390 on maintenance and renewal

Rats were trained in Context A on the seek-take task. After 3 d of stable baseline responding, SCH 23390 (*n* = 13) or saline (*n* = 10) was given before self-administration sessions in the BLA for 3 d (Phase 1). Subsequently, rats given SCH 23390 were given saline and rats given saline were given SCH 23390 (Phase 2). On discovering an order effect in the first cohort, only rats in the Sal/SCH group received continued seek-take training 2 d (Days 10 and 11) and then were moved to Context B for two additional days (Days 12 and 13). Subsequently, rats were moved back to Context A (Day 14). Four animals lost patency during the course of the experiment and was excluded. Throughout the context changes, rats had continued drug access and did not have any intra-BLA injections.

#### Experiment 1B: Effects of post-session BLA SCH 23390 on maintenance

A separate naïve group of rats (*n* = 6) were cannulated, catheterized, and trained on the seek-take task as described above. After stable baseline was reached, rats were injected with SCH 23390 after cocaine self-administration sessions for 3 d, followed by a return to baseline with no intra-BLA injection for 2 d.

#### Experiment 2: Effects of BLA or CeA SCH 23390 on maintenance

Two groups of rats were catheterized and cannulated in the BLA (*n* = 7) or the CeA (*n* = 7) and trained on the seek-take task for an equivalent number of days. The BLA and CeA cannulated groups did not differ in the level of drug exposure during acquisition or in the rate of acquisition of the seek-take procedure. After 3 d of baseline, both groups were given SCH 23390 before the self-administration sessions for 3 d then given saline for three additional days.

#### Experiment 3: Effects of BLA or CeA SCH 23390 on maintenance and renewal

Two groups of rats were catheterized, cannulated in the BLA (*n* = 10) or the CeA (*n* = 14), and trained on the seek-take task for an equivalent number of days in Context A (context identity was counterbalanced between Contexts A and B). After 3 d of baseline, rats were given SCH 23390 (*n* = 17) or saline (BLA/CeA: *n* = 7) for 4 d. On the 3rd day of SCH 23390 or saline, all groups were moved to Context B (counterbalanced). On the 4th day of SCH 23390 or saline, all groups were moved back to Context A. Both groups had drug access throughout the experiment.

### Microinjection procedures

During injection days, dummy cannulas were removed and injection cannulas attached to 1-µl Hamilton syringes were inserted bilaterally into guide cannulas. A total volume of 0.3 µl of either saline (0.9%) or D1 antagonist (SCH 23390) was injected at a rate of 0.1 µL/min using a microinjection pump (Chemyx) ∼4 min before a self-administration session. The microinjecter was left in place for 1 min to allow for diffusion.

### Drugs

SCH 23390 (Tocris) was dissolved in saline (0.9% NaCl) to a concentration of 2 µg/0.3 µl. Cocaine hydrochloride was dissolved in saline to a concentration of 2 mg/ml.

### Conditioned place aversion (Experiment 4)

#### Apparatus and general behavioral methods

Place conditioning followed the unbiased apparatus and procedures outlined by Cunningham et al. (2006) and used previously by our lab to assess BLA function (Bernardi et al., 2009). The apparatus consisted of identical boxes (48 × 16 × 20 cm) in sound attenuating chambers that were custom fabricated (McCarthy Manufacturing) with a camera mounted 46 cm above each box and an IR light source mounted 43 cm along the long wall of each box. Video was collected and analyzed using ANY-maze behavioral tracking software (Stoelting). The floor of each box consisted of interchangeable halves made of a grid floor or a hole floor that were 26.5 × 20.5 cm raised on a 2.5-cm acrylic frame. The grid floor consisted of 2.3-mm stainless-steel rods mounted 13 mm apart. The hole floor consisted of a perforated stainless-steel plate with 13-mm round holes. There were three phases in the experiment over 5 d: pretest (one session: 30 min), conditioning (three sessions: 60 min), and testing (one session: 30 min). Rats were given intracranial surgery as described above using the CeA and BLA coordinates 7 d before the first phase and were randomly assigned to one of four groups: hole + (*n* = 5), hole – (*n* = 5), grid + (*n* = 5), and grid – (*n* = 5). Rats were tracked using the center point of the animal.

#### Pretest

Rats were placed into the apparatus consisting of a half grid floor and a half hole floor, with the position of the floors counterbalanced within each subgroup to test for a natural preference. After 30 min, rats were returned to their home cages.

#### Conditioning

Twenty-four hours after pretest, rats were microinjected with saline (0.9%; CS−) or with D1 antagonist (2.0 μg/.3uL; CS+) into the BLA or CeA as described above. Injectors were left in place for 1 min following injection to allow for diffusion. Immediately after, rats were placed into the apparatus with one floor type either hole or grid. The order of the CS+ and CS– was counterbalanced each day within each subgroup. Conditioning sessions were 60 min each day for 3 d.

#### Testing

Twenty-four hours after the last conditioning session, rats were placed into the apparatus consisting of a half hole floor and a half grid floor, with the position of the floor counterbalanced within each subgroup. Preference was determined by time spent on the CS+ side and CS–, with a middle zone of 18 cm (the approximate length of a rat) excluded to account for when rats were both on the hole and grid floors. After 30 min, rats were returned to their home cages. Rats were not injected on test day.

### Statistical analyses

Seek and take lever instrumental responding in self-administration sessions were analyzed using a two-way ANOVA with session, time within session, or a context as a repeated factor, drug treatment as the between-subjects factor, and seek and take presses, or time to complete each session as the dependent variable. Bonferroni-adjusted, two-tailed, paired-samples *t* tests or independent *t* tests were used for *post hoc* tests where appropriate. All data were represented as the mean ± SEM. Statistical tests where *p* ≤ 0.05 was considered significant and statistics are reported in the statistical table (Table 1).

**Table 1.** Summary of statistical analysis

Figure	Test	*n*	Comparison	Statistical significance
1*B*	Repeated measures ANOVA + Bonferroni	D1 = 13, saline = 10	(Seek) day × group phase 1	*p* = 0.05
			(Seek) day × group phase 2	*p* = 0.001
1*D*			(Take) day × group phase 1	*p* = 0.01
			(Take) day × group phase 2	*p* = 0.00005
1*B*,*D*			(Seek + take) *post hoc* day 2 D1 vs saline	*p* < 0.008
			(Seek + take) *post hoc* day 3 D1 vs saline	*p* < 0.001
1*C*	ANOVA	D1 = 13, saline = 10	Saline vs D1 day 1 phase 1	*p* = 0.007
			Saline vs D1 day 2 phase 1	*p* = 0.0004
			Saline vs D1 day 3 phase 1	*p* = 0.00001
			Saline vs D1 day 1 phase 2	*p* = 0.953
			Saline vs D1 day 2 phase 2	*p* = 0.002
			Saline vs D1 day 3 phase 2	*p* = 0.013
1*C*	Repeated measures ANOVA + Bonferroni	D1 = 13, saline = 10	Day × group phase 1	*p* = 0.664
			Day × group phase 2	*p* = 0.017
1*E*	ANOVA	D1 = 6, saline = 6	Seek	*p* = 0.986
			Time	*p* = 0.206
2*B*	*t* test	D1 = 13, saline = 10	Day 3 SAL/SCH vs SCH/SAL	*p* = 0.02
2*B*	Paired *t* test	*n* = 6	Day 11 vs day 12	*p* = 0.00037
			Day 13 vs day 14	*p* = 0.01651
3*A*	Repeated measures ANOVA + Bonferroni	CeA = 6, BLA = 7	Session × group	*p* = 0.049
			BLA vs CeA day 1	*p* = 0.038
			BLA vs CeA day 2 + 3	p > 0.363
3*B*	Repeated measures ANOVA + Bonferroni	CeA = 8, BLA = 9	Group × room	p = 0.003
			Context B: CeA vs BLA	*p* = 0.0001
3*B*	One-way ANOVA	Sal = 7	baseline, context shift, original context	*p* = 0.6
4*A*	Repeated measures ANOVA + Bonferroni	Sal = 10, BLA = 11, CeA = 5	Main effect test	*p* < 0.0001
			Main effect group	*p* < 0.00006
			Day × group	*p* = 0.05
			BLA vs saline 45 min	*p* < 0.01
			CeA vs saline 15 and 30 min	*p* ≤ 0.05
			Saline vs BLA and CeA 60 min	*p* > 0.1
4*B*	One-way ANOVA	Sal = 10, BLA = 11, CeA = 5	BLA vs saline	*p* = 0.000007
			BLA vs CeA	*p* = 0.001
			CeA vs saline	*p* = 0.669
4*D*	ANOVA	Sal = 10, BLA = 11, CeA = 5	Main effect test	*p* = 0.001
			Main effect group	*p* = 0.971
			Group × test	*p* = 0.792

#### Time bin analysis

Drug seek responses were binned and averaged every minute and a group raster plot was analyzed and constructed with ElTemps software (A. Diez Noguera, University of Barcelona, Spain).

## Results

### Dopamine D1 receptor antagonism in the BLA progressively reduces intravenous cocaine self-administration

**Figure 1.**
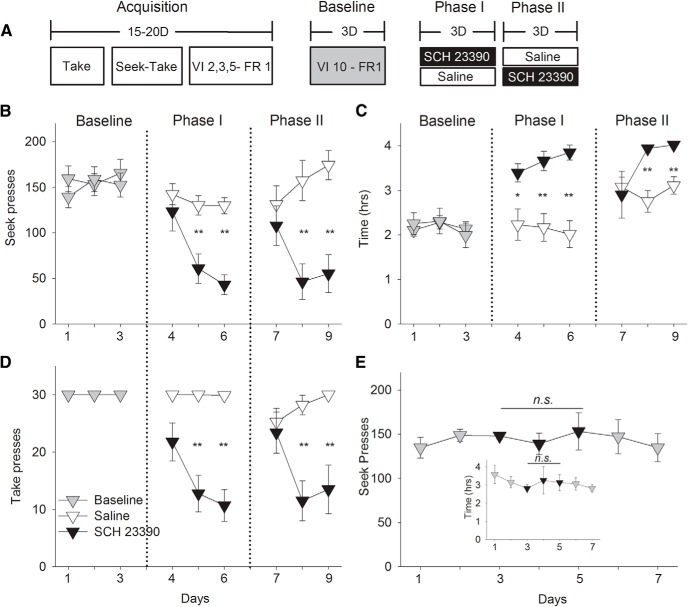
Intra-BLA D1 blockade by SCH 23390 progressively decreases cocaine seeking and taking behavior and increases the time to complete each session in Experiment 1. ***A***, Experimental timeline showing the seek-take procedure from acquisition, baseline, Phase 1, and Phase 2 in Experiment 1A. After the acquisition period of 15–20 d, 3 d of stable baseline were recorded before SCH 23390 (*n* = 13, 2.0 μg/0.3 µl) or saline (*n* = 10, 0.3 µl) was injected before each session (Phase 1). Subsequently, SCH 23390-treated rats were given saline and saline-treated rats were given SCH 23390 for three additional days (Phase 2). ***B***, SCH 23390 (black filled) administered before each session progressively decreased cocaine seeking. *Post hoc* analysis revealed differences between saline on Days 2 and 3 of drug treatment (*p* < 0.05). ***C***, SCH 23390 treatment resulted in an increase in the time to complete each session compared to saline controls (white unfilled). *Post hoc* analysis revealed differences between saline and drug treatment (*p* < 0.05). ***D***, SCH 23390 decreased cocaine taking behavior on Days 2 and 3 of drug treatment (*p* < 0.05). ***E***, In Experiment 1B, SCH 23390 administered after each session (black filled; *n* = 6) did not alter subsequent cocaine seeking and taking behavior or time to complete each session, relative to two sessions of baseline (gray filled) before and after SCH 23390 treatment (inset; *p* > 0.1). Error bars indicate SEM; **p < 0.01, *p < 0.05. n.s., non significant.

Two groups of rats were trained on a V10-FR1 seek-take chain schedule of reinforcement to procure intravenous cocaine rewards in Experiment 1A (Fig. 1*A*). After stable baseline of seek and take responses, rats were given the D1 antagonist SCH 23390 (2 µg/0.3 µL) or saline injections into the BLA before self-administration sessions for three consecutive days (Phase 1). Subsequently, conditions were reversed such that the SCH 23390-treated group was given saline and the saline-treated group was given SCH 23390 for three additional days (Phase 2). In both conditions, BLA D1 receptor blockade progressively decreased cocaine seeking (Fig. 1*B*) and taking (Fig. 1*D*) behavior compared to the saline-treated group (ANOVA: Phase 1 seek: day × group: *F*_(2,36)_ = 3.150, *p* = 0.05, take: day × group: *F*_(2,36)_ = 5.21, *p* = 0.01; Phase 2 seek: day × group: *F*_(2,24)_ = 9.46, *p* = 0.001; take: day × group: *F*_(2,24)_ = 15.34, *p* = 0.00005). *Post hoc* analysis revealed a significant group difference on seek and take lever presses on Day 2 (*p* < 0.008) and Day 3 (*p* < 0.001) of drug treatment and was accompanied with an increase of time to complete each session (ANOVA: Phase 1: *F*_(1,22)_ = 10.467, *p* = 0.007, Day 2: *F*_(1,20)_ = 17.79, *p* = 0.0004, Day 3: *F*_(1,19)_ = 35.285, *p* = 0.00001, Phase 2: Day 1: *F*_(1,18)_ = 0.004, *p* = 0.953, Day 2: *F*_(1,16)_ = 13.586, *p* = 0.002, *F*_(1,15)_ = 8.174, *p* = 0.013; repeated measures ANOVA: Phase 1: day × group: *F*_(2,36)_ = 0.415, *p* = 0.664; Phase 2: day × group: *F*_(2,28)_ = 4.708, *p* = 0.017; Fig. 1*C*). Post-session delivery of SCH 23390 did not alter drug seeking or taking responses or time to complete the session in Experiment 1B, suggesting that the D1 blockade weakened responding for cocaine without affecting memory consolidation (seek: *F*_(6,18)_ = 0.154, *p* = 0.986; time: *F*_(6,18)_ = 1.59, *p* = 0.206; Fig. 1*E*).

**Figure 2.**
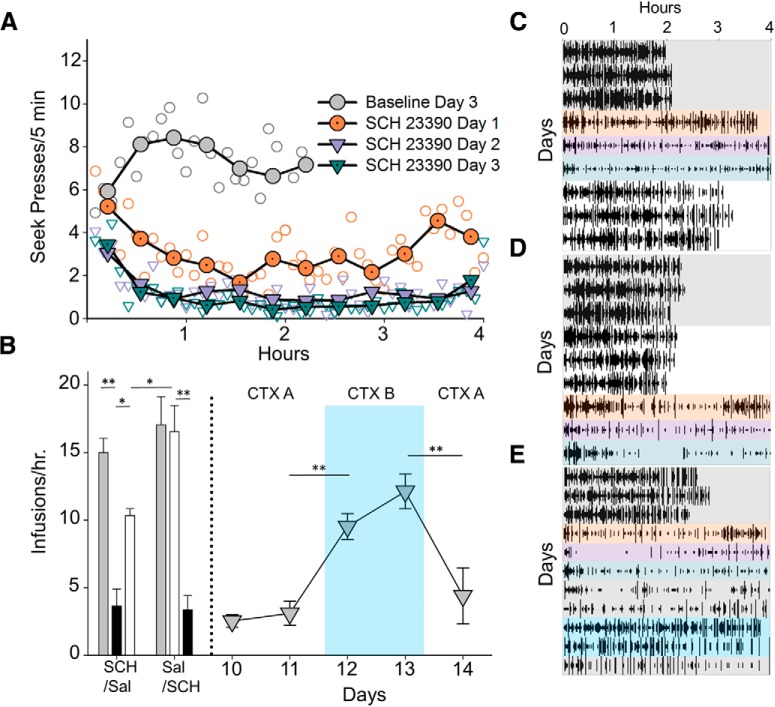
Time bin analysis of responding during 3 d of intra-BLA administration of D1 blockade by SCH 23390 and effects on contextual renewal in Experiment 1. ***A***, Time bin analysis of responding during 3 d of intra-BLA administration of D1 blockade by SCH 23390. Results show while rats exhibited a steady rate of responding during the last day of baseline (charcoal), rats showed a greater U-shaped pattern of responding on Day 1 (desert orange) versus Day 2 (purple) and Day 3 (turquoise). ***B***, left panel, Number of infusions per hour on the last day of baseline (gray), SCH 23390 (black), and saline (white) treatment for both groups. Rate analysis revealed an order effect between the SCH/Sal group and the Sal/SCH group (*p* < 0.05). Rats in the Sal/SCH group (*n* = 6) were maintained under baseline conditions and continued to show a persistent suppression of the rate of responses in the training context for 2 d (CTX A: Days 10 and 11), exhibited a contextual renewal in a novel context (CTX B: Days 12 and 13), and a resuppression of responding in the training context (paired *t* test: *p* < 0.01; CTX A: Day 14). Group raster plot of seek presses of (***C***) SCH/Sal group, (***D***) Sal/SCH group, and (***E***) the Sal/SCH renewal group during baseline (charcoal), Day 1 (desert orange), Day 2 (purple), and Day 3 (teal) of SCH 23390 and saline (white) treatment. In addition, ***E*** shows the return to baseline in context A for 2 d (charcoal), test in Context B for 2 d (blue), and a final session in Context A (charcoal); **p < 0.01, *p < 0.05.

A time bin analysis of seek presses revealed a distinct pattern of responding during drug treatment. Figure 2*A* shows within-session responding during baseline and three SCH 23390 sessions. Figure 2*C–E*shows raster plots of the subgroups that received SCH 23390 before saline (SCH/Sal; Fig. 2*C*), saline before SCH 23390 (Sal/SCH; Fig. 2*D*), and saline before SCH 23390 followed by context change (Fig. 2*E*). While during baseline sessions, rats exhibited continuous pressing activity that lasted on average 2.06 h (SD 0.67), BLA D1 blockade resulted in a U-shaped pattern of responding typically lasting the entire session with responding highest at the beginning and end of each session (Fig. 2*A*,*C–E*). On Days 2 and 3 of drug treatment, both groups showed a greater suppression of responses, and continued to show a U-shaped pattern of responding. Counterbalancing drug treatment revealed that rats given SCH 23390 first (SCH/SAL; Fig. 2*B*,*C*) continued to show a persistent decrease in the rate of cocaine infusions after they were switched to saline, compared to rats given SCH 23390 after saline [SAL/SCH (Fig. 2*B*,*D*); SAL/SCH vs SCH/SAL: *t*_(11)_ = 2.68, *p* = 0.02].

To assess the persistence of the SCH 23390-induced reduction in cocaine self-administration, rats given saline then SCH 23390 were maintained on the seek-take procedure with no intra-BLA injections for 2 d in the drug acquisition context (CTX A: Days 10–11), moved to a distinct context for two additional days (CTX B: Days 12–13), and then placed back into the original context (CTX A: Day 14; Fig. 2*B*, right panel), all with continued access to cocaine. Subsequent rate analysis revealed that rats increased the number of infusions/hour when moved to Context B (paired *t* test: *t*_(5)_ = 8.5, *p* < 0.01), then decreased the rate of responding when returned to Context A (paired *t* test: *t*_(5)_ = 3.97, *p* < 0.01; Fig. 2*B*,*E*), suggesting that SCH 23390 delivery resulted in a persistent, context-specific attenuation of self-administration.

### The time course of the suppressive effects of D1 receptor antagonism in the BLA and CeA differs during maintained self-administration

**Figure 3.**
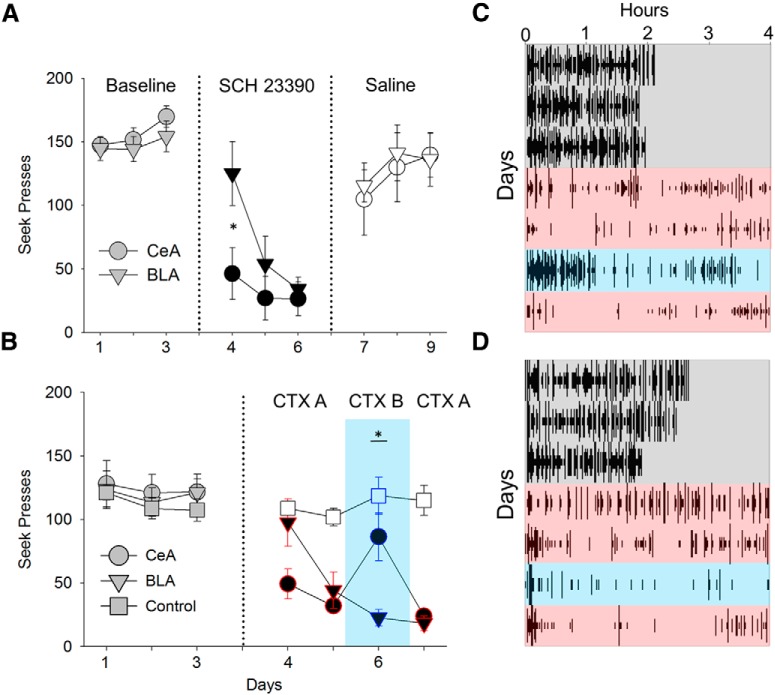
Different sensitivities of suppression between the CeA and BLA on cocaine-seeking behavior and effects on contextual renewal during concurrent D1 blockade in Experiments 2 and 3. ***A***, While D1 blockade (dark filled) in the CeA (*n* = 6) rapidly decreased cocaine-seeking behavior, D1 blockade in the BLA (*n* = 7) progressively decreased cocaine-seeking behavior. Saline treatment recovered cocaine-seeking behavior in both the CeA and BLA group. ***B***, Consistent with the results found in Figure 2*A*, D1 blockade in the BLA (*n* = 8) and the CeA (*n* = 9) differentially decreased cocaine-seeking behavior over 2 d in the training context (Context A; red outline) while saline controls (white unfilled: BLA/CeA, *n* = 7) did not change. On the 3rd day of D1 blockade or saline, groups were moved to Context B (cyan), where the CeA group, but not the BLA group, increased cocaine-seeking behavior (*p* < 0.05). On the 4th day of D1 blockade, groups were returned to Context A, where both groups showed a suppression of cocaine-seeking behavior. Group raster plot of seek lever presses of the CeA group (***C***) and BLA group (***D***) during baseline (charcoal), SCH 23390 treatment in context A (red), SCH 23390 treatment in context B (cyan), and SCH 23390 treatment back in Context A (red); **p < 0.01, *p < 0.05.

There is much evidence that the CeA and BLA play distinct roles in reward learning (Parkinson et al., 2000). To investigate the suppressive effects of D1 blockade in the CeA and BLA, two groups of rats were catheterized and cannulated in the CeA (*n* = 6) or the BLA (*n* = 7) and trained on the seek-take task as described above. Consistent with Experiment 1, D1 BLA blockade in Experiment 2 progressively decreased cocaine self-administration through 3 d of treatment (Fig. 3*A*). However, D1 CeA blockade reduced cocaine self-administration rapidly in the first session and persisted through additional sessions of SCH treatment (session × group: *F*_(2,20)_ = 3.52, *p* = 0.049). *Post hoc* analysis revealed a significant group difference on Day 1 (*p* = 0.038). Both the BLA and CeA groups recovered their drug-seeking responses when subsequently treated with saline, mirroring the residual D1 blockade effects by SCH 23390 we observed in the first experiment.

### D1 receptor antagonism in the CeA or BLA suppresses cocaine self-administration, but the CeA-induced suppression reverses with a context change

In Experiment 3, rats were catheterized and cannulated in the CeA (*n* = 8) or BLA (*n* = 9) and were trained on the seek-take task and given SCH 23390 or saline for 4 d after 3 d of baseline in a ABA context renewal procedure (Fig. 3*B*). Similar to Experiment 2, both groups decreased drug seeking during 2 d of SCH 23390 delivery in Context A with the CeA group showing greater sensitivity to D1 blockade on Day 1. However, when moved to a novel Context B, the CeA group, but not BLA group, increased cocaine self-administration during concurrent D1 blockade (ANOVA: group × room *F*_(2,39)_ = 6.92, *p* = 0.003, context B: CeA vs BLA, *p* = 0.0001; for group raster plot, see Fig. 3*C*). During the 4th day of D1 blockade, the BLA and CeA group were returned to Context A where both groups again showed a suppression of cocaine self-administration. The saline group (*n* = 7) did not differ in responding through baseline, saline treatment during the context shift, and saline treatment back in the original context (one-way ANOVA: *F*_(3,45)_ = 0.54, *p* = 0.6). These results suggest D1 activity in the BLA, but not the CeA, to be necessary for context-mediated renewal of drug-seeking behavior under continued drug access conditions.

### Conditioned place aversion to SCH 23390 in the CeA versus BLA

**Figure 4.**
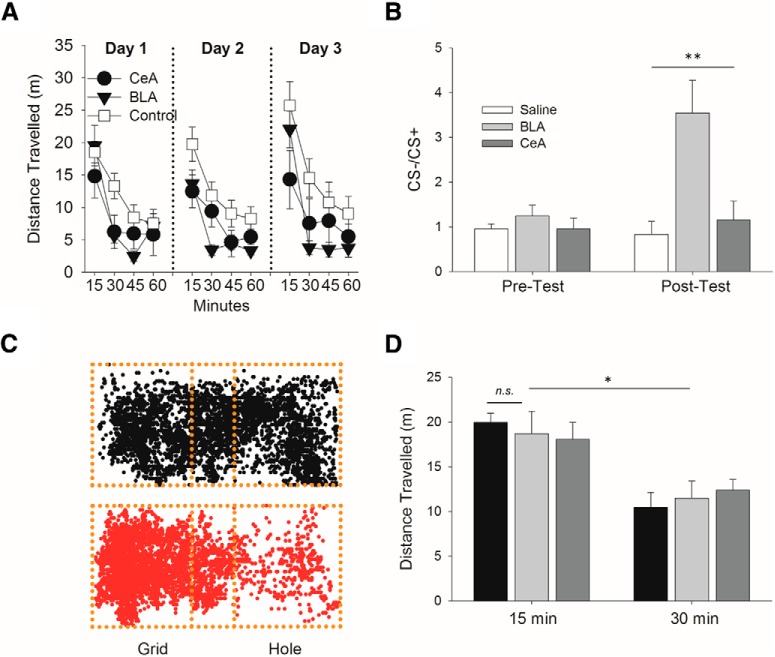
Intra-BLA, but not intra-CeA, D1 blockade by SCH 23390 for 3 d results in a conditioned place aversion in Experiment 4. ***A***, Saline (*n* = 10), BLA (*n* = 11), and CeA (*n* = 5) groups decreased their distance traveled within each session of conditioning, but the BLA and CeA group given SCH 23390 (black filled) had a larger decrease then saline controls (unfilled; *p* ≤ 0.05). ***B***, Saline, BLA, and CeA groups did not have a preference for the grid or hole floors during the pre-test. However, after 3 d of conditioning, the BLA, but not the saline or CeA groups, showed a significant conditioned place aversion (CS–/CS+: ratio of time spent in non-conditioned side vs conditioned side) during the *post hoc* test (*p* < 0.05). ***C***, Representative tracking plots of rats during *post hoc* test for saline (top, black) and intra-BLA SCH 23390 (red, bottom) groups injected during training on the hole floor. A mid-zone compartment of 18 cm in length (the approximate length of the body of an animal) was used in the analysis to account for when the animal was both on the grid and hole floors. ***D***, While all three groups decreased their distance traveled during the *post hoc* test, there were no differences between groups (*p* > 0.9); **p < 0.01, *p < 0.05. n.s., non significant.

To investigate the rewarding or aversive effects of intra-amygdalar D1 blockade, SCH23390 or saline was microinjected (2 µg/0.3 µl/side) into the BLA or CeA before each place conditioning session for 3 d to mirror drug treatment during the self-administration experiments (Experiment 4). Both SCH groups and the saline group decreased the total distance traveled during each conditioning day (main effect of test *F*_(3,84)_ = 23.093, *p* < 0.0001, main effect of group: *F*_(2,84)_ = 10.780, *p* < 0.00006). However, the BLA and CeA groups given SCH 23390 showed a larger decrease in distance traveled compared to saline controls (day × group *F*_(4,168)_ = 2.326, *p* = 0.05; Fig. 4*A*). Collapsed across days, *post hoc* analysis revealed that the BLA group differed from the saline group at the 30- and 45-min mark (*p* < 0.01) but not the 15-min mark (*p* = 0.15), while the CeA group differed from the Saline group at the 15- and 30-min mark (*p* ≤ 0.05), but not at the 45 min mark (*p* = 0.13). Both the CeA and the BLA group did not differ from saline controls at the 60 min mark (*p* > 0.1). On test day in drug free conditions, the BLA, but not CeA group, showed a significant conditioned place aversion compared to saline controls (one-way ANOVA: *F*_(2,45)_ = 5.529, *p* = 0.007, BLA vs saline: *p* = 0.000007, BLA vs CeA: *p* = 0.001, CeA vs saline: *p* = 0.669; Fig. 4*B*,*C*). During the *post hoc* test, all three groups significantly decreased their distance traveled from the 15- to 30-min test point (ANOVA: test: *F*_(1,38)_ *p* = 12.423, *p* = 0.001), but did not differ from each other (group: *F*_(2,38)_ = 0.029, *p* = 0.971, group × test: *F*_(2,38)_ = 0.238, *p* = 0.792; Fig. 4*D*).

**Figure 5.**
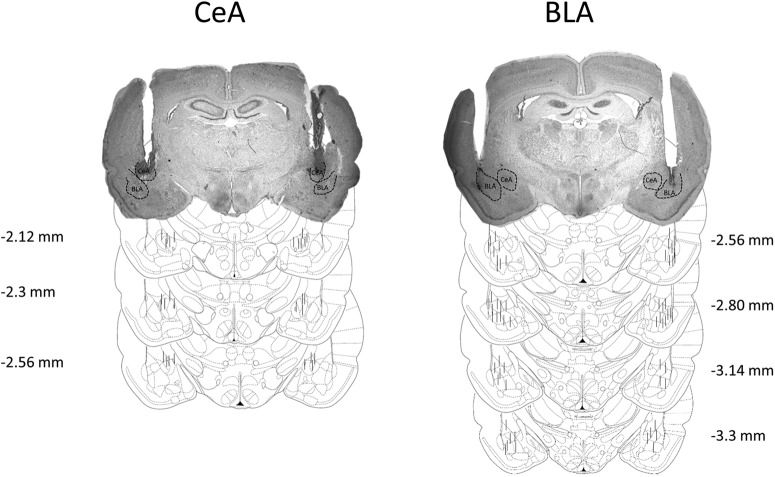
Reconstruction of cannula placement for CeA and BLA groups with representative CeA and BLA photomicrographs. Each charcoal line represents the tip of the infusion cannula that protruded (∼0.75 mm) from the guide cannula.

## Discussion

These experiments found that dopamine D1 receptor antagonism by SCH 23390 in the BLA or CeA disrupted stable cocaine self-administration. The speed with which these effects occurred, as well as their persistence with a change in context, differed between the two regions. BLA blockade of D1 receptors induced a gradual U-shaped pattern of suppression within and between cocaine self-administration sessions (Experiments 1 and 2). This effect persisted when SCH 23390 was removed with continued cocaine access, but reversed with continued access in a new context (Experiment 1). When SCH 23390 continued to be delivered, the suppression persisted into a new context (Experiment 3). In contrast, D1 antagonism in the CeA caused a rapid decrease in cocaine self-administration (Experiments 2 and 3) that reversed with a shift to a new context, even in the continued presence of SCH 23390. Together, these results suggest that D1 receptors in the BLA and CeA contribute to maintained cocaine self-administration, but may do this through mechanisms that are differentially mediated by contextual associations.

Within and between sessions in the same context, D1 antagonism in the BLA or CeA resulted in attenuated self-administration under continued access procedures. In the CeA, this attenuation was rapid. In the BLA, the attenuation was more gradual, with a progressive U-shaped suppression of drug seeking responses to intra-BLA D1 blockade within a session, suggesting that SCH 23390 competed with reinforcing properties of cocaine self-administration. Previous research has shown that striatal levels spike at 30 min and last up to 4 h with a subcutaneous injection of SCH 23390 (Hietala et al., 1992). Although the half-life of SCH 23390 in the amygdala is not known, the drug-seeking data suggest suppression to begin 30 min into the session, while recovery of responses begins near the end of the 4-h session. However, with repeated sessions with BLA D1 blockade, the U-shaped suppression of drug seeking became more pronounced, consistent with learned suppression of responding over the course of repeated sessions with the antagonist. The gradual effect that emerged over days is consistent with other studies of the BLA, which have found a delayed effect of BLA lesions on cue-induced reinstatement with no immediate effect on maintained cocaine self-administration (Meil and See, 1997). These and other findings suggest the importance of multiple long-term behavioral tests to reveal amygdala function in drug reward (Baxter and Murray, 2002; Pizzimenti et al., 2017).

### Context changes reveal differences in persistence of D1 antagonism effects

The suppressive effect was reversed when SCH 23390 was removed during continued access conditions in the same context or when the context was changed under conditions without SCH 23390. When continued access continued in the presence of SCH 23390 in a different context, the suppressive effect persisted in the BLA, but reversed in the CeA. This finding of weakened renewal during continued access in the BLA is consistent with other studies that have found that D1 blockade before a nonreinforced test of cue- or context-induced reinstatement weakens responding (Alleweireldt et al., 2002; Crombag and Shaham, 2002; Hamlin et al., 2006, 2007). The differences in the speed with which BLA and CeA effects occurred and their persistence across contexts is unlikely due to effects on motor function because SCH 23390 in either region resulted in similar decreases in locomotion in Experiment 4, but differences in the speed with which instrumental responding decreased in Experiments 1–3. One caveat between the experiments is that the effect of D1 antagonism on locomotion in the CPA experiment was in naive rats, whereas the rats in the self-administration experiments had a history of cocaine. Perhaps the magnitude of the locomotor effects of D1 antagonism in the BLA may vary with a history of cocaine self-administration. However, post-session administration of SCH 23390 was without effect, consistent with an interaction between the antagonist and the experience during the self-administration session, again suggesting that blockade in the amygdala alters learning processes to weaken instrumental responding.

The decreases in drug seeking responses to intra-BLA D1 blockade, slow recovery to saline, and renewal in a novel context is similar to changes that occur during extinction, post-extinction reacquisition, and contextual renewal (Bouton, 1986; Bouton et al., 2012; Bouton and Todd, 2014). In our Experiment 3, however, these changes occurred in the presence of continued reinforcement of the response, meaning that the renewal that occurred with a change in context was not entirely dependent on nonreinforcement. Other studies have found that renewal occurs after counterconditioning (Peck and Bouton, 1990) and that renewal-like effects occur under other conditions in which the organism attends to the context to disambiguate conflicting information about the stimulus-outcome relation (Nelson and Callejas-Aguilera, 2007; Rosas and Aguilera-Callejas, 2007). Thus, contextual renewal can be applied to cases outside of the typical reinforcement followed by nonreinforcement situations and there is an emerging literature, suggesting that contexts can modulate specific response-outcome associations (Trask and Bouton, 2014).

### Possible mechanisms of D1 suppression on drug seeking and taking

There are several mechanisms through which the suppressive effects of D1 antagonism could occur. A very general mechanism would be a disruption of activity in the amygdala that may operate through D1 or D2 receptor mechanisms. A number of studies have shown that a single administration of a D1 (Caine et al., 1995; Alleweireldt et al., 2006) but not D2 antagonist (See et al., 2001) in the BLA has disrupted cocaine self-administration and administration of a D1 agonist in the BLA has been found to increase cocaine-seeking behavior (Mashhoon et al., 2009), consistent with the present results. Because cocaine self-administration increases DA release in the BLA and the nucleus accumbens (NAc; Hurd and Pontén, 2000) and D1 antagonism in the BLA has been found to block the excitability of DA on BLA projection neurons, it is possible that amygdalar blockade by SCH 23390 may decrease the activity of D1 projection neurons and alter DA tone in the Nac to disrupt the primary reinforcing properties of cocaine. These types of mechanisms will clearly require further investigation.

The finding in Experiment 4 that D1 antagonism in the BLA induced a place aversion suggests that cues associated with SCH 23390 may acquire aversive properties. This is consistent with other studies that have found that D1 antagonism blocks cocaine CPP (Nazarian et al., 2004) and can induce a conditioned place aversion when administered in the NAc (Shippenberg et al., 1991). These aversive properties may overshadow the rewarding properties of cocaine, causing self-administration to decrease independent of context, but when the antagonist is removed, the behavior recovers quickly. It also is possible that SCH 23390 altered the rewarding value of cocaine itself, leading to a weakened reinforcer that failed to maintain responding. However, SCH 23390 in the CeA did not induce a place aversion, although it resulted in an immediate disruption of responding for cocaine, suggesting a dissociation between the aversive effects of the compound itself and its effects on maintained responding for cocaine.

BLA neurons have been found to respond to both appetitive and aversive stimuli (Belova et al., 2007) and many studies have found that DA signaling in the amygdala is involved in aversive learning paradigms (Fadok et al., 2010; Darvas et al., 2011; Abraham et al., 2016a,b; Lee et al., 2017). A recent study found that population coding of stimulus and outcome neural ensembles in the BLA underwent learning-induced changes through the recruitment of unresponsive BLA neurons before learning (Zhang and Li, 2018), suggesting that subsets of BLA neurons may become engaged by one aspect of learning while other neurons become engaged by other aspects of the same learning process. Thus, it is possible that neurons within the BLA encode context-dependent (conditioned value) and context-independent (unconditioned value) features, which revealed themselves as stronger and weaker contextual renewal, respectively, in our experiments.

Given that dopamine has been shown to play a critical role in reinforcement processes, the decrease in drug seeking responses to intra-amygdalar D1 blockade suggests an interference of DA signaling that may represent outcome expectations. Many studies have shown that overexpectation of reward magnitude can lead to a decrease in appetitive conditioned responding (Lattal and Nakajima, 1998; Rescorla, 2007) and have implicated the CeA in Pavlovian overexpectation effects, with some evidence that the BLA may be involved with instrumental contingencies (Haney et al., 2010; Holland, 2016). Perhaps D1 antagonism in the amygdala alters the expected value of the reward and thereby creates a persistent overexpectation effect that weakens some aspect of the association between responding and cocaine.

In line with a role in Pavlovian-instrumental interactions, intra-BLA D1 blockade has been shown to block cue-induced instrumental responding (See et al., 2001). In Pavlovian-to-instrumental transfer tasks, BLA lesions result in deficits in specific cue-induced instrumental behavior, while CeA lesions show deficits in general non-specific cue-induced instrumental behavior (Corbit and Balleine, 2005). These results have been interpreted to suggest the BLA to encode outcome value with specific sensory features, while the CeA to encode the general representation of motivational processes. Analogously, in devaluation tasks, BLA lesion animals decrease instrumental responding to both non devalued and devalued outcomes, suggesting a general devaluation across different cues to remain intact, while CeA lesions continued to show selective devaluation (Corbit and Balleine, 2005). In line with these results, intra-BLA D1 blockade may cause a devaluation of the reinforcer that continues to suppress responses across context shifts. In contrast, intra-CeA blockade might enable the BLA to show specific cue-induced instrumental behavior and increase responding on a contextual renewal test. These findings also are consistent with work from Thrailkill and Bouton (2015) showing that specific goal-directed responding can transfer across contexts (such as occurred with our BLA suppression), but stimulus-response learning is sensitive to context change (such as occurred with our CeA suppression). Clearly there are multiple accounts that warrant further study, but our findings suggest additional ways that the BLA and CeA may make distinct contributions to value representation, adding evidence for a parallel rather than serial model of amygdala function (Balleine and Killcross, 2006).

### A model of relapse under continued access conditions

Many models of drug relapse incorporate abstinence and relapse under non-reinforced conditions to test how drug-associated cues can alter motivated behavior. While these results have been invaluable to understanding how drug associated cues can motivate instrumental responding, the therapeutic challenges with forced abstinence models are that social, environmental, and emotional events can robustly trigger a relapse of drug seeking and taking behavior. Alternate therapeutic approaches that decrease drug seeking responses under safe drug access conditions need to be explored involving pharmacological blockade and chemical aversion therapy (Dole et al., 1966; Frawley and Smith, 1990; Elkins et al., 2017). Recently, additional animal models of relapse under continued access conditions, such as punishment-based and conflict-based models have been used to mirror other human clinical abstinence and relapse behaviors (Marchant et al., 2013). In these continued access conditions, BLA inactivation has been found to have opposite effects on contextual renewal after punishment compared to extinction (Pelloux et al., 2018). Additionally, studies have found that amygdala neurons can serve alternate functions under different conditions. For example, the BLA and CeA are both engaged during contextual renewal (Knapska and Maren, 2009), but inactivation of the BLA and CeA can have different effects on acquisition, extinction, and expression of reward-related behaviors (Kruzich and See, 2001). Many studies have shown that the BLA and the CeA have distinct anatomic and functional circuits that modulate DA efflux in the NAc (Everitt et al., 1999; Phillips et al., 2003; Shiflett and Balleine, 2010). Given these results, more research into how the different subdivisions of the amygdala can alter the motivational and associative processes of reward may hold the key to therapeutic interventions that are successful and resistant to relapse.

## Synthesis

Reviewing Editor: Yavin Shaham, NIDA-IRP/NIH Decisions are customarily a result of the Reviewing Editor and the peer reviewers coming together and discussing their recommendations until a consensus is reached. When revisions are invited, a fact-based synthesis statement explaining their decision and outlining what is needed to prepare a revision will be listed below. The following reviewer(s) agreed to reveal their identity: Nathan Holmes, Jeffrey Grimm.

The reviewers found significant merit in the study but raised several minor concerns that should be addressed with text revisions. I enclose below the individual comments of the reviewers, and in addition to their comments, also address the minor comments below:

1. Abstract and other places in the text: Separate numbers from letter when reporting measurement units (2 µg/.3µL should be 2 µg/0.3 µL)

2. Significant section: the first sentence is unclear, please revise. Also, please change ‘crucial insight’ to ‘insight’

3. Methods and other places in the text: replace ‘animals’ with ‘rats’ when referring to the experimental subjects

4. Discussion: please add 2-3 informative sub-headings to orient the readers of the topics being discussed

REVIEWER 1

OVERALL

The paper examines the effects of BLA and CeA D1 receptor antagonism on the maintenance of cocaine self-administration (seeking and taking) in rats. The major findings are that BLA and CeA infusions of the D1 receptor antagonist, SSCH23390, have common effects on the maintenance of cocaine self-administration in the training context, but dissociable effects on the maintenance of cocaine self-administration in a novel context. When rats were tested in the training context, infusions of SCH23390 into either region suppressed cocaine self-administration; but this suppression was reversed when the SCH23390 infusions were discontinued. When rats were tested in a novel context, cocaine self-administration was again suppressed following a SCH23390 infusion into the BLA, but unaffected following a SCH23390 infusion into the CeA. Together, these results were taken to imply that SCH23390 infusions have both context-independent and context-dependent effects when infused into the BLA and CeA respectively: infusions into the BLA suppressed cocaine self-administration in both the training and novel contexts, whereas infusions into the CeA only suppressed cocaine self-administration in the training context. They are discussed with respect to the differing roles of the BLA and CeA in processing different aspects of a reward (e.g., sensory and motivational).

The paper is well-written, the experiments are well-designed, and the major inferences are supported by appropriate statistical analyses. The findings are likely to be of interest to a broad readership. I do, however, have a few minor points that should be addressed in revisions prior to acceptance.

CONCEPTUAL

Perhaps the discussion on pages 19-20 might consider that the BLA infusion of SCH23390 affected the portion of responding controlled by the drug value (i.e., the action), whereas the CeA infusion of SCH23390 affected the portion of responding controlled by the context (i.e., the S-R control over responding by the context) or the various response-contingent cues that accompany delivery of the drug.

In relation to the BLA results: it would also be worth considering that, if a BLA infusion of SCH23390 reduces the drug value (and thereby, the portion of responding controlled by the drug value), it could generate a persistent over-expectation effect (as the experienced drug is not as pleasurable as the expected drug) that is conditioned to the context and underlies the context-specific inhibition of self-administration in Experiment 1.

Introduction

Page 3 para 2: “Although these studies have demonstrated dopaminergic function in the amygdala to be critical for conditioned stimuli to integrate value representation with appropriate instrumental responses...” Disturbances of cue-induced reinstatement (produced by manipulations of dopamine signalling or other) do not necessarily imply some effect of the treatment on “integration” of value representation with instrumental responses: e.g., the treatment could simply affect retrieval of the Pavlovian cue-drug association. Consider clarifying or deleting.

Page 4 para 1: “In these models, the basolateral (BLA) and the central nucleus of the amygdala (CeA) were found to have opposite effects on context induced relapse compared to the extinction reinstatement model (Marchant, et al., 2018).” What were the opposite effects of BLA and CeA manipulations on context-induced relapse in the two cases that are noted? Given the arguments for studying these regions, and the return to these arguments in the discussion, it would be worth providing a very short description of the data suggesting that they play (i) opposite roles in context-induced relapse (as a function of the protocol), and (ii) distinct roles in studies of reward learning.

Method

Were the animals housed individually or in a group?

Page 7 para 2: “Upon discovering an order effect, a subset of animals in the Sal/SCH group (n = 6) received continued seek-take training for 2 days (Days 10 and 11) and then moved to Context B for 2 additional days (Days 12 and 13).” What happened to the remaining 17 animals in this experiment? Did they lose patency in their catheters? I'm just wondering why the Sal/SCH rats (or a subset of them) were not subjected to the same treatment that is described at the bottom of page 12.

Re Experiment 4, Conditioned Place Aversion (CPA): Did rats exhibit a natural preference for one side of the apparatus used to assess the CPA? It would be worth explicitly stating this, one way or the other, in the text for this section. It would also be worth noting in the discussion of these results that the effects of the D1 antagonist were assessed in rats that are naïve to cocaine. It seems reasonable to suppose that the likelihood of a conditioned place aversion would be magnified in rats with a history of self-administration (such as that used in Experiments 1-3).

Results

Was there an effect of the D1 receptor antagonist on cocaine taking independently of its effect on cocaine seeking? Or, alternatively phrased: upon gaining access to the taking lever, was there a difference between the two groups in how quickly they initiated drug taking, or not? This could be addressed by expressing the taking responses as a proportion of the seeking responses, and assessing group-differences in these data. However it comes out, the results of this analysis would provide more information regarding the role of amygdala D1 receptor activation in cocaine self-administration.

Were the numbers of days required to achieve 3 days of stable performance (baseline) the same for BLA and CeA rats, and the same across the different experiments? Is it possible that the BLA and CeA rats differed in their levels of drug exposure at the point of the critical manipulations, and could this then explain at least some of the differences between the BLA and CeA rats? I don't expect that there were any such differences, or that they explain a large portion of the effects. Nonetheless, it would be worth stating these details so that the reader need not be distracted by such considerations.

Please check the stats reported in the brackets at the end of page 11: there are some inconsistencies in the usage of brackets and in the labelling of critical statistics within the brackets.

Page 12 Para 1: “Post-session delivery of SCH 23390 did not alter drug seeking and taking responses or time to complete the session, suggesting that the D1 blockade weakened responding for cocaine without affecting memory consolidation (Seek: F6,18 = 0.154, p = 0.986, Time: F6,18 = 1.59, p = 0.206) (Figure 1E). Can the authors please provide procedural details regarding the effects of post-training D1 antagonist infusions on responding: e.g., was this assessed in a naïve group of animals?

How does the data presented in Figure 2B relate to the data presented in Figure 1D? It appears as though there is a modest difference in take presses for rats injected with saline in phase I or II (comparison of the white triangles in the middle and right panels), which translates into a larger difference in numbers of infusions (2B) between the same groups. Is this correct?

On page 13, it is stated: “Post hoc analysis revealed a significant group difference on Day 1 (p = 0.038), but not on Days 2 and 3 (p > 0.363).” If the authors maintain this post-hoc approach to the data, the Bonferroni correction that they described in the section on statistical analyses would mean that the difference on day 1 is not significant. Here and elsewhere, a trend analysis would be more appropriate (linear, quadratic), which would obviate the need for post-hoc tests like the ones used here. I'm not suggesting a complete change in approach to the statistics, but rather, an appropriate qualification on the claimed significance of this particular result. Alternatively, simply omit the post-hoc tests on days 2 and 3, which appear to be unnecessary for the point that the authors wish to make (more rapid suppression in the CeA rats relative to the BLA rats).

On page 13, it is stated: “Both the BLA and CeA groups gradually recovered their drug-seeking responses when subsequently treated with saline mirroring the residual D1 blockade effects by SCH 23390 we observed in the first experiment.” Is this supported by a statistical analysis: was there a linear increase in responding across days 7-9?

Discussion

Page 17 Para 1: “The differences in the speed with which BLA and CeA effects occurred and their persistence across contexts is unlikely due to effects on motor function because SCH 23390 in either region resulted in similar decreases in locomotion in Experiment 4, but differences in the speed with which instrumental responding decreased in Experiments 1-3.” As noted above, it should be acknowledged in the discussion that the locomotor effects of the D1r antagonist may vary with a history of cocaine self-administration.

Page 19 Para 1: I couldn't quite follow the statement here: “Thus it is possible that neurons within the BLA encode context-dependent (attenuated conditioned value) and context-independent (attenuated unconditioned value) features, which reveal themselves as stronger and weaker contextual renewal, respectively. Please revise.

Are the results specifically due to antagonism of dopamine receptors in either region (or are they due to general effects of the SCH23390 infusion on neuronal activity in the BLA and CeA), and are they specifically observed following antagonism of D1 receptors in either region (or would infusions of a D2 receptor antagonist generate a similar pattern of results)? This deserves some minor comment in the discussion.

REVIEWER 2

This is an interesting manuscript that will add to the understanding of the complex relationship between amygdalar subregions and responding directed towards primary and secondary reinforcement. Limitations of the report are that the study had many moving parts including measures of seeking and taking, repeated drug administration, and manipulation of context. This may relate to some of the comments and criticisms of previous reviewers regarding the limited rationale development and interpretation of results. That being said, there are strengths including assessing both central and basolateral amygdala, and following behavior for several days post-test. In addition, including the assessment of aversive effects of amygdalae SCH23390 was important, as were later additions of vehicle-treated subjects. The discussion is appropriately tempered. There are definitely several aspects of this study that deserve follow up, in particular the idea that appetitive information is processed in parallel by the amygdalar subregions, instead of in serial. In addition, the authors might consider findings of inactivation of the basolateral amygdala that did not have immediate effects on cocaine taking (Meil and See 1997; Grimm and See 2000). The Meil and See paper might be of particular interest as rats with lesioned basolateral amygdala responded for cocaine with no obvious impairment for many days. Perhaps blockade of a discrete D1 receptor-mediated pathway is critical for the “devaluation” observed in the present study.
